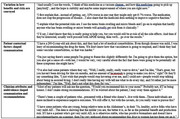# Characterizing Clinician Communication with Patients about Lecanemab: A Qualitative Study of Clinicians across Seven Academic Medical Centers

**DOI:** 10.1002/alz70858_096332

**Published:** 2025-12-24

**Authors:** Ayush Thacker, Anna L Parks, Daniel Dohan, Liliana A Ramirez Gomez, Kim G Johnson, Christine S Ritchie, Sachin J Shah, Joanna Paladino

**Affiliations:** ^1^ Mongan Institute Center for Aging and Serious Illness, Division of Palliative Care and Geriatric Medicine, Massachusetts General Hospital, Boston, MA, USA; ^2^ University of Utah, Salt Lake City, UT, USA; ^3^ University of California, San Francisco, San Francisco, CA, USA; ^4^ Massachusetts General Hospital, Harvard Medical School, Boston, MA, USA; ^5^ Duke University, Durham, NC, USA; ^6^ Massachusetts General Hospital, Boston, MA, USA; ^7^ Harvard Medical School, Boston, MA, USA

## Abstract

**Background:**

Lecanemab, an anti‐amyloid monoclonal antibody, modestly slows cognitive decline in early Alzheimer's disease but may cause adverse events, including amyloid‐related imaging abnormalities due to edema (ARIA‐E) or hemorrhage (ARIA‐H). A small percentage of ARIA‐E and ARIA‐H cases may be disabling or fatal. As lecanemab becomes available, understanding clinician communication of its benefits and risks to patients and caregivers is crucial. This qualitative study investigates clinician communication of lecanemab's risks and benefits to support patient and caregiver decision‐making.

**Methods:**

We conducted semi‐structured interviews with clinicians who prescribe anti‐amyloid therapy at seven academic medical centers. An interdisciplinary research team used hybrid inductive‐deductive thematic analysis.

**Results:**

27 clinicians completed interviews (Women [*n* = 17], White [*n* = 19], Neurologists [*n* = 20]). Three preliminary themes emerged. First, clinicians used varied approaches to describe the therapy's benefits and risks. They used analogies to explain lecanemab's mechanisms and discussed statistical outcomes from the CLARITY‐AD clinical trial, often stating ‘this is not a cure.’ While all clinicians communicated the risks, they differed in how much they emphasized or de‐emphasized their clinical impact, particularly ARIA. Second, patient contextual factors shaped communication. Clinicians personalized conversations based on patients’ comorbidities, caregiver support, treatment hopes and fears, and eligibility criteria fit. Third, For example, while clinicians honor patients’ choices to pursue treatment, many do not routinely ‘recommend’ it (but may recommend against it given particular patient factors).

**Conclusions:**

Clinicians at the forefront of lecanemab treatment use a variety of communication approaches to discuss benefits and risks. These insights can guide future interventions to improve communication and decision‐making for lecanemab.